# Meta-analysis of factors influencing depression in cervical cancer patients

**DOI:** 10.3389/fpubh.2025.1657690

**Published:** 2025-11-03

**Authors:** Rong Wang, Bang Liu, Si-Wen Wei, Yi-Ran Liu, Yi Zhong, Li Han, Huai-Qing Luo

**Affiliations:** ^1^Department of Pathophysiology, School of Basic Medical Sciences, Hunan Normal University, Changsha, Hunan, China; ^2^Changsha Maternal and Child Health Care Hospital Affiliated to Hunan Normal University, Changsha, Hunan, China; ^3^Jishou University School of Medicine, Jishou, Hunan, China; ^4^Hunan Provincial University Key Laboratory of the Fundamental and Clinical Research on Functional Nucleic Acid, Changsha Medical University, Changsha, Hunan, China; ^5^Department of Physiology, School of Basic Medical Sciences, Hunan Normal University, Changsha, Hunan, China

**Keywords:** cervical cancer, depression, influencing factors, meta-analysis, case-control study

## Abstract

**Background:**

Depression in cervical cancer (CC) patients concurrently compromises disease management and quality of life. However, significant discrepancies persist among existing studies regarding the determinants of depression in this population worldwide. To address this gap, this study employs meta-analysis to systematically identify and synthesize the contributing factors to depression among cervical cancer patients.

**Aim:**

To provide evidence-based references for mitigating depression risk among cervical cancer patients.

**Methods:**

Literature was searched in databases including CNKI, Wanfang, VIP, CBM, Web of Science, PubMed, and EMBASE from their inception until March 2025. The literature was screened, selected, quality assessed, and data extracted and analyzed. Meta-analysis was conducted using Revman 5.4 and Stata 18 software, with odds ratios (OR) and their 95% confidence intervals (CI) as the observed indicators.

**Results:**

A total of 1,108 articles were retrieved, with 15 articles ultimately included in the analysis. The results indicate that low educational attainment (OR = 3.25, 95% CI: 2.02–5.22), age ≥45 years (OR = 1.67, 95% CI: 1.09–2.55), inter-household monthly income disparity (OR = 3.06, 95% CI: 1.87–5.00), advanced tumor stage (OR = 1.99, 95% CI: 1.28–3.11), low social support (OR = 2.48, 95% CI: 1.95–3.16), moderate to severe pain (OR = 2.86, 95% CI: 1.76–4.65), limited disease awareness (OR = 2.58, 95% CI: 1.88–3.55), and undergoing hysterectomy (OR = 4.69, 95% CI: 3.03–7.24) are significant risk factors for depression in cervical cancer patients.

**Conclusion:**

The occurrence of depression in cervical cancer patients is influenced by multiple factors. Healthcare professionals and family members should conduct comprehensive assessments of patients' conditions to implement targeted prevention and intervention measures, thereby enhancing the psychological wellbeing of patients.

**Systematic review registration:**

https://inplasy.com/, identifier: INPLASY202560039.

## 1 Introduction

Cervical cancer refers to the malignant tumor arising from the transformation zone, which is the area at the junction between the squamous epithelium of the ectocervix and the columnar epithelium of the endocervical canal (1). The common malignant tumor poses a significant threat to women's health and has become a major global public health issue (2). According to the GLOBOCAN 2022 global cancer statistics, cervical cancer ranks eighth in incidence worldwide (662,000 cases, accounting for 3.3%) and ninth in mortality (349,000 cases, accounting for 3.6%) (3, 4). According to online statistics from the National Cancer Institute, it is estimated that in 2025, 13,360 new cases and 4,320 deaths will occur. The entire process from diagnosis to treatment completion may adversely impact patients' mental health with both immediate and prolonged effects. Studies have reported that the prevalence of depression among cervical cancer patients in China ranges from 31.5 to 76.3%, significantly exceeding levels observed in the country's general population and surpassing those reported among cancer patients in other countries (5–9). The occurrence of depression in cervical cancer patients may hinder their active engagement in cancer treatment and affect their adherence to therapy and adoption of healthy lifestyles. This could potentially accelerate cancer progression, thereby shortening survival time. The impact of cancer on patients with depression is significant. Studies have shown that depression can predict the mortality rate of cancer patients, with those experiencing depressive symptoms having a 25% higher mortality rate (10). Additionally, depression has a significant impact on the immune system. Research indicates that it can directly stimulate the production of pro-inflammatory cytokines, which, in turn, affects the development of various diseases, including cancer (11). Depression can also accelerate cancer progression by weakening immune responses, leading to a significant reduction in patients' survival periods. These findings collectively suggest that depression plays an important role in the course and treatment of cancer.

Currently, research findings on the factors influencing depression among cervical cancer patients are inconsistent both domestically and internationally (6, 12–25). Therefore, this study employs a meta-analysis approach to synthesize recent research on this issue, systematically evaluating the factors influencing depression in cervical cancer patients. The findings aim to provide reference for clinical practice and effectively guide healthcare professionals in the early screening and management of high-risk populations.

## 2 Materials and methods

### 2.1 Search strategy

#### 2.1.1 Literature sources

Literature published in databases from their inception to March 2025 was searched in CNKI, Wanfang, VIP, CBM, Web of Science, PubMed, and EMBASE to identify all potentially eligible studies.

#### 2.1.2 Search strategy

Chinese search terms included “cervical cancer,” “depression,” and “influencing factors” as subject headings, combined with corresponding free-text terms such as “cervical carcinoma,” “cervical malignant tumors,” “cervical intraepithelial neoplasia,” “invasive cervical cancer,” “depression and related factors,” and “risk factors.” English-language search utilized “Cervical Cancer,” “Depression,” and “Risk Factor” as subject headings, along with corresponding free-text terms such as “Carcinoma of Cervix,” “uterine cervix cancer,” “malignant tumor of cervix,” “Carcinoma of the cervix,” “Malignant tumor of the cervix,” “Depressive Disorder,” “depressive state,” “depressed,” “depressive,” as well as “Influence factor” and “Relative risk.” Terms of the same category were linked using the logical operator “OR,” while terms from different categories were connected with “AND.” Relevant references from the retrieved literature were also reviewed.

### 2.2 Inclusion and exclusion criteria

#### 2.2.1 Inclusion criteria

① Study types: cohort studies, case-control studies, or cross-sectional studies. ② Study subjects: diagnosed cervical cancer patients. ③ Study content: analysis of factors influencing depression in cervical cancer patients. ④ Depression screening: utilization of the Self-Rating Depression Scale (SDS), Hospital Anxiety and Depression Scale (HADS), Beck Depression Inventory (BDI), or Hamilton Depression Rating Scale (HAMD). ⑤ Completeness of data: literature must provide complete data, appropriate statistical methods, and directly provide odds ratios (OR) with corresponding 95% confidence intervals (95% CI), or sufficient data to calculate these.

#### 2.2.2 Exclusion criteria

① Study types: not cohort studies, case-control studies, or cross-sectional studies. ② Inability to extract valid outcome data: studies that do not provide valid outcome data. ③ Literature types: animal experiments, non-clinical literature, reviews, systematic evaluations, and meta-analyses. ④ Duplicate literature.

### 2.3 Quality assessment

Different scoring standards were applied based on the type of literature. For case-control studies, we utilized the Newcastle-Ottawa Scale (NOS) (26, 27) recommended by the Cochrane Collaboration. This scale includes eight evaluation items, with a total score of 9, specifically divided into: selection of study population (four items, 4 points), comparability between groups (one item, 2 points), and outcome measurement (three items, 3 points). The evaluation results are presented in Table 1.

**Table 1:** Quality evaluation of case-control study.

**Author/year**	**Study population selection**	**Intergroup comparability**	**Outcome measurement**	**Total points**
Song Hongxia (2021) (12)	4	0	3	7
Zhu Yuping (2024) (13)	4	2	2	8
Wang Yan (2020) (14)	4	2	2	8
He Leying (2022) (16)	4	2	2	8
Tang Jianan (2021) (17)	4	2	2	8
Sun Shujuan (2013) (18)	4	2	3	9
You Lina (2019) (19)	4	0	2	6
Li Jianxiang (2013) (6)	4	0	2	6
Ke Yinghua (2019) (20)	4	2	2	8
Wang Xuan (2019) (21)	4	2	2	8
Lao Chengming (2020) (22)	4	2	3	9
Zhu Jing (2024) (23)	4	0	2	6
Zou Shuqian (2021) (24)	4	2	2	8

For cross-sectional studies, we employed the quality assessment tool from the Joanna Briggs Institute (JBI) Evidence-Based Healthcare Center (28). This tool comprises nine items covering aspects such as sampling methods, study subjects, data collection, and analysis methods, with each item rated as “Yes,” “No,” “Unclear,” or “Not Applicable.” The assessment results are presented in Table 2.

**Table 2:** Quality evaluation of cross-sectional study.

**Author/year**	**1**	**2**	**3**	**4**	**5**	**6**	**7**	**8**	**9**	**Total score**
Yang Yingzhen (2017) (15)	√	√	√	√	√	√	√	√	√	100%
Soo Hyun Kim (2010) (25)	×	√	√	√	√	√	√	√	?	78%

√: yes; × : no; ?: unclear.

1 Was the sample frame appropriate to address the target population?

2 Were study participants sampled in an appropriate way?

3 Was the sample size adequate?

4 Were the study subjects and the setting described in detail?

5 Was the data analysis conducted with sufficient coverage of the identified sample?

6 Were valid methods used for the identification of the condition?

7 Was the condition measured in a standard, reliable way for all participants?

8 Was there appropriate statistical analysis?

9 Was the response rate adequate, and if not, was the low response rate managed appropriately?

The assessment process was conducted independently by two researchers. In cases of disagreement, a third party was consulted for arbitration to reach a consensus.

### 2.4 Data extraction

Based on the requirements of the study, the following data were extracted from the full text: authors, year, region, type of literature, sample size, depression assessment scales, and related influencing factors, as shown in Table 3.

**Table 3:** General information of the included.

**Author/year**	**Population origin**	**Study type**	**Sample size**	**Depression screening scales**	**Risk factors**
Song Hongxia (2021) (12)	Jiangsu	Case-control study	130	SDS	1,3
Zhu Yuping (2024) (13)	Jiangsu	Case-control study	1,623	HADS	1,3,4,5
Wang Yan (2020) (14)	Henan	Case-control study	150	BDI	4
Yang Yingzhen (2017) (15)	Anhui	Cross-sectional study	217	BDI	3,4
He Leying (2022) (16)	Zhejiang	Case-control study	90	SDS	1,3,4
Tang Jianan (2021) (17)	Guangdong	Case-control study	200	SDS	5,6,7
Sun Shujuan (2013) (18)	Hunan	Case-control study	196	SDS	2,3,5,6,7
You Lina (2019) (19)	Jiangsu	Case-control study	368	SDS	1,3,6,7,8
Li Jianxiang (2013) (6)	Guangxi	Case-control study	500	SDS	1,3,6,7,8
Ke Yinghua (2019) (20)	Guangdong	Case-control study	289	SDS	1,4,6,7,8
Wang Xuan (2019) (21)	Beijing	Case-control study	48	SDS	1,3
Lao Chengming (2020) (22)	Zhejiang	Case-control study	94	SDS	2,3,5,6,7
Zhu Jing (2024) (23)	Shanxi	Case-control study	80	HAMD	3,5
Zou Shuqian (2021) (24)	Guangdong	Case-control study	200	HAMD	1,3,4,8
Soo Hyun Kim (2010) (25)	Korea	Cross-sectional study	1,328	HADS	2,3

1. Low education attainment; 2. Age ≥45 years; 3. Inter-household monthly income disparity; 4. Tumor stage (advanced stage); 5. Low social support; 6. Moderate-to-severe pain; 7. Limited disease awareness; 8. Hysterectomy.

### 2.5 Data analysis

Data analysis was conducted by using Review Manager 5.4 and Stata 18. The odds ratio (OR) and its 95% confidence interval (CI) were utilized as the outcome measures. Heterogeneity among studies was evaluated by using the *Q* statistic and *I*^2^ statistic; when *P* > 0.1 and *I*^2^ < 50%, it indicated that the heterogeneity between studies was not significant, and a fixed-effect model was employed. Conversely, a random-effects model was used if the heterogeneity was significant. Publication bias was assessed by using Begg's test. Sensitivity analysis was performed by examining the magnitude of differences between the fixed-effect and random-effects model data. *P* < 0.05 was considered statistically significant.

## 3 Results

### 3.1 Literature search results

**Figure 1:**
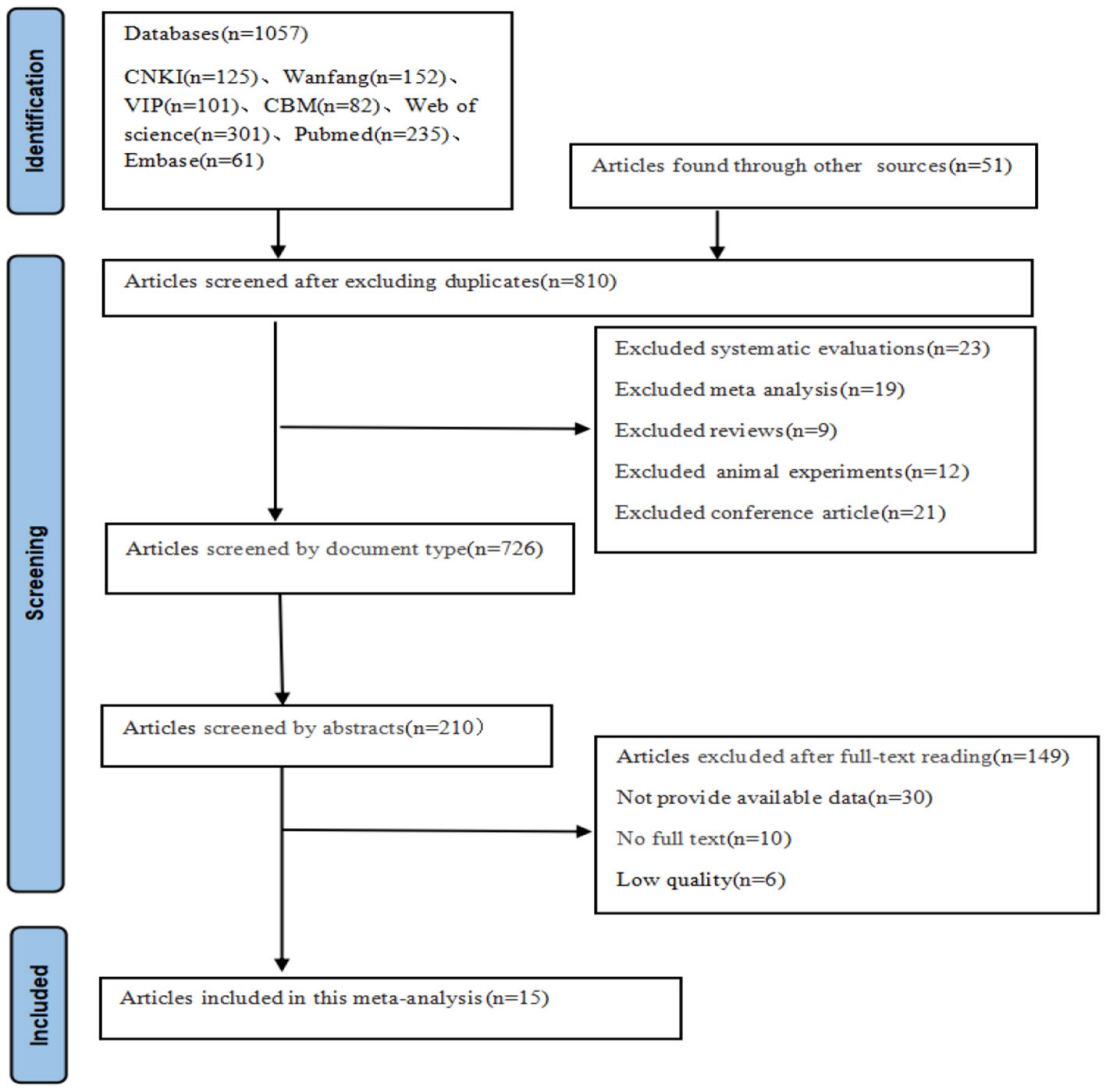
Literature screening flowchart.

A total of 1,108 articles were collected from seven databases. After a thorough screening process, 15 articles were included, comprising 14 in Chinese and one in English. The literature selection process is illustrated in Figure 1.

### 3.2 Meta-analysis results

#### 3.2.1 Low educational attainment

**Figure 2:**
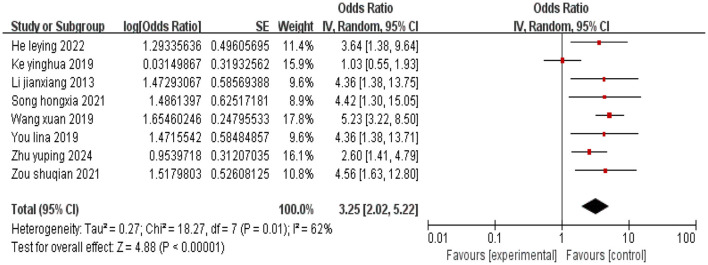
Forest plot of the impact of low education level on depression in cervical cancer patients.

A total of eight studies were included in the analysis, and heterogeneity among the studies was observed (*I*^2^ = 62%). A random effects model was employed, and the combined effect size indicated that a low educational attainment in cervical cancer patients is associated with the occurrence of depression (*P* < 0.05), as shown in Figure 2.

#### 3.2.2 Age ≥45 years

**Figure 3:**
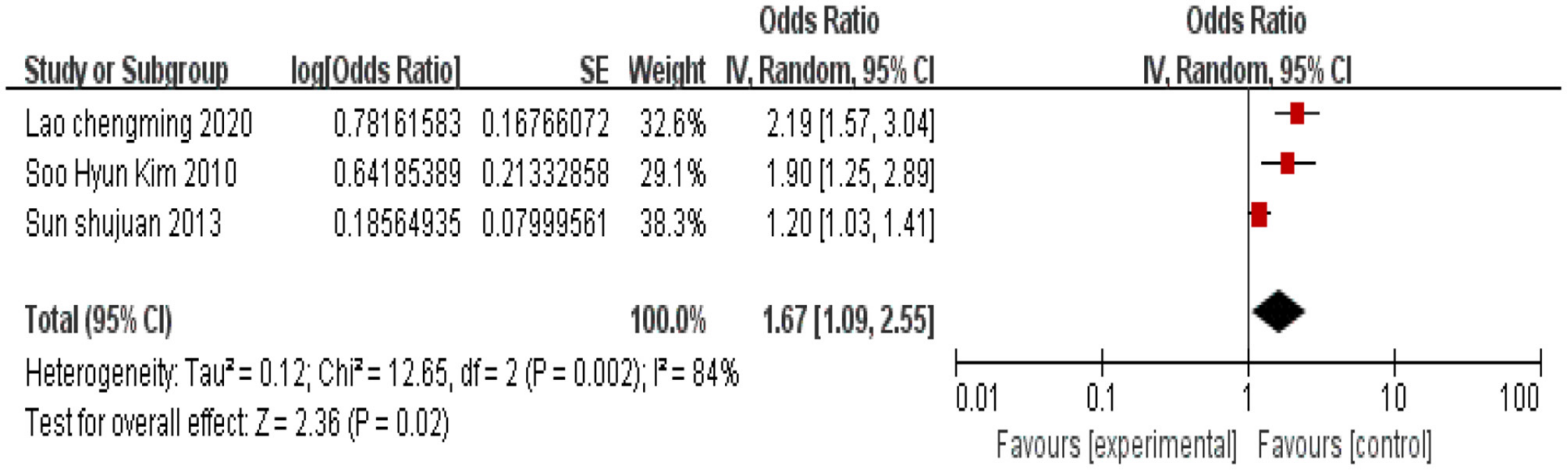
Forest plot of the impact of age ≥45 years on depression in cervical cancer patients.

A total of three studies were included, and heterogeneity among the studies was observed (*I*^2^ = 84%). Using a random effects model, the combined effect size indicated that cervical cancer patients aged ≥45 years are associated with an increased incidence of depression (*P* < 0.05), as shown in Figure 3.

#### 3.2.3 Inter-household monthly income disparity

**Figure 4:**
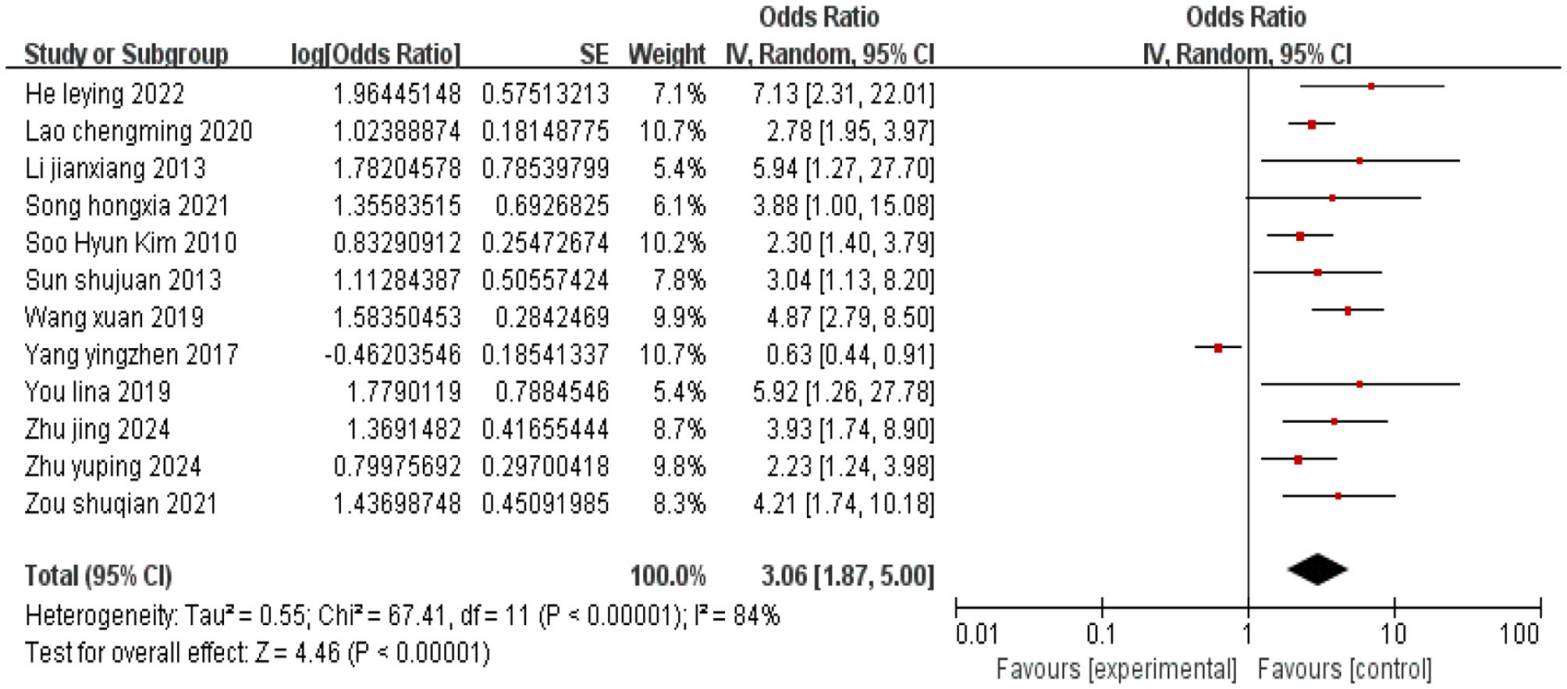
Forest plot of the impact of inter-household monthly income disparity on depression in cervical cancer patients.

A total of 12 studies were included, and heterogeneity was observed among the studies (*I*^2^ = 84%). Using a random effects model, the combined effect size indicated a correlation between family monthly income disparity and the occurrence of depression in cervical cancer patients (*P* < 0.05), as shown in Figure 4.

#### 3.2.4 Tumor stage (advanced stage)

**Figure 5:**
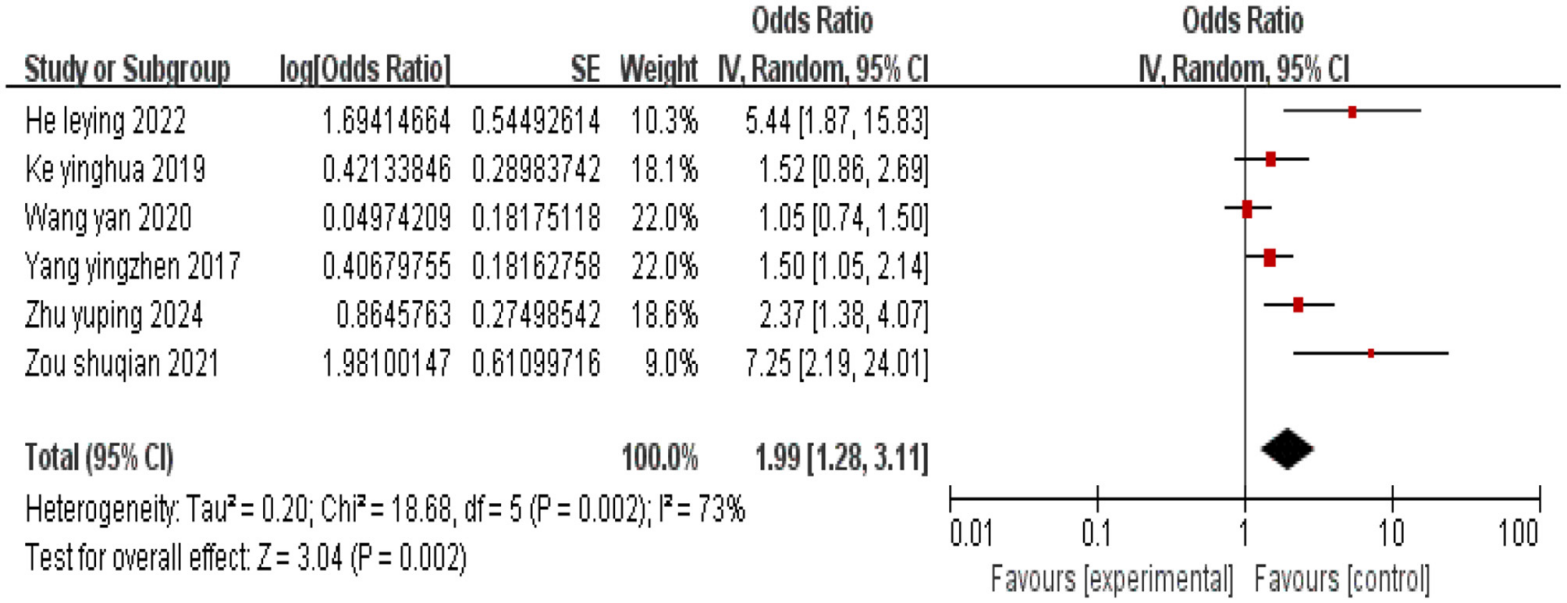
Forest plot of the impact of advanced tumor stage on depression in cervical cancer patients.

A total of six studies were included, and heterogeneity among the studies was observed (*I*^2^ = 73%). Using a random effects model, the combined effect size indicated that cervical cancer patients with advanced tumor stage are associated with an increased incidence of depression (*P* < 0.05), as shown in Figure 5.

#### 3.2.5 Low social support

**Figure 6:**
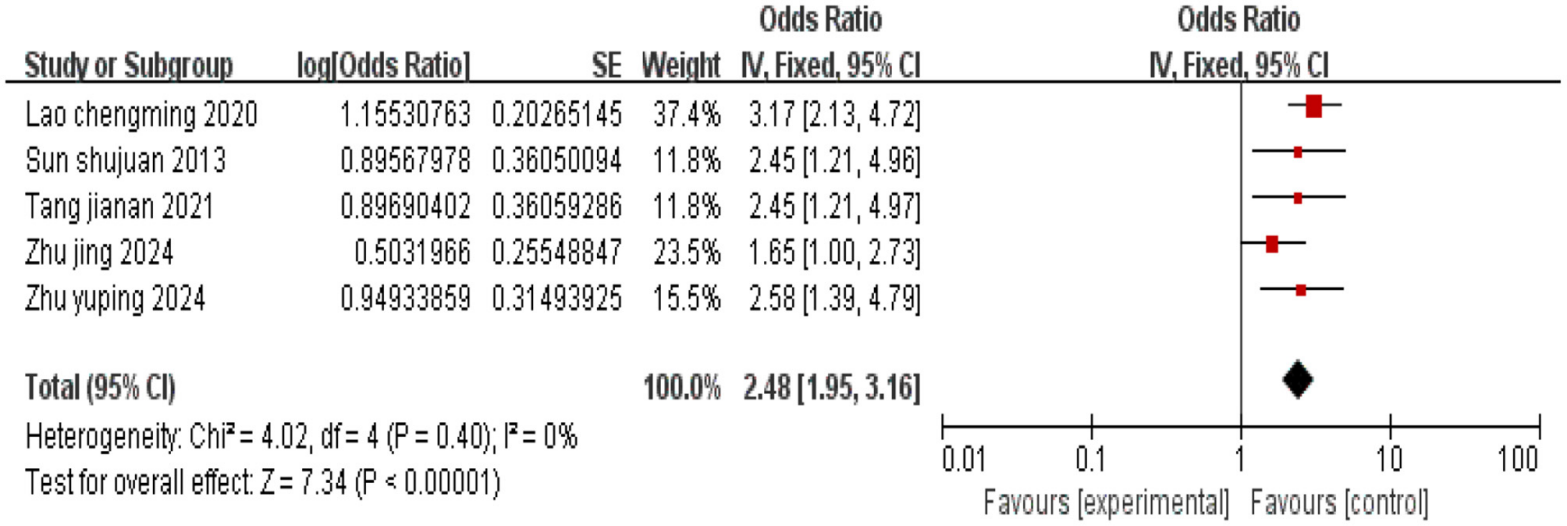
Forest plot of the impact of low social support on depression in cervical cancer patients.

A total of five studies were included, and there was no heterogeneity among the studies (*I*^2^ = 0%). Using a fixed-effects model, the combined effect size indicated that low social support is associated with depression in cervical cancer patients (*P* < 0.05), as shown in Figure 6.

#### 3.2.6 Pain severity (moderate or higher)

**Figure 7:**
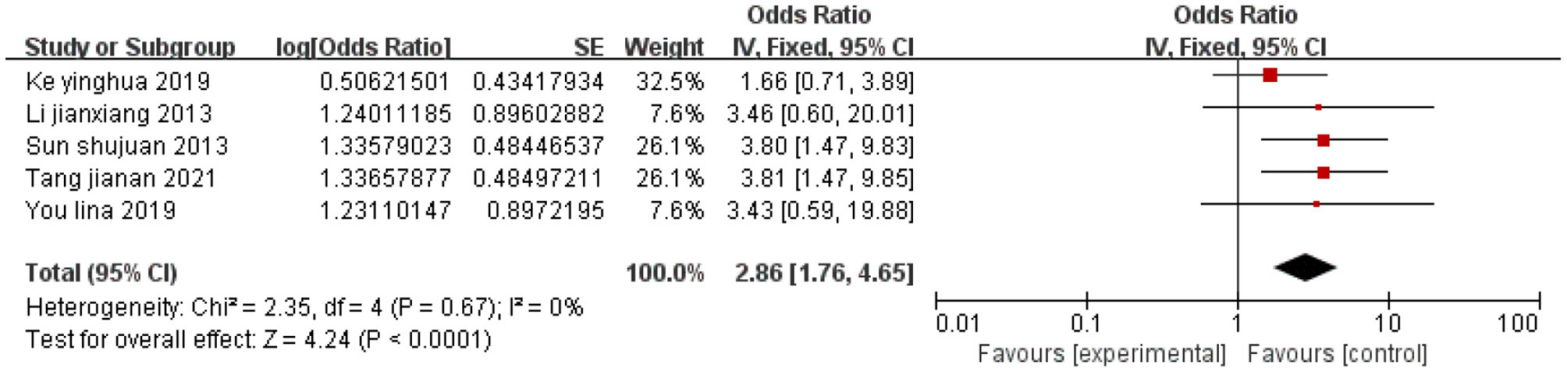
Forest plot of the impact of moderate-to-severe pain on depression in cervical cancer patients.

A total of five studies were included, and there was no heterogeneity among the studies (*I*^2^ = 0%). Using a fixed-effects model, the combined effect size indicated that moderate or higher pain severity in cervical cancer patients is associated with depression (*P* < 0.05), as shown in Figure 7.

#### 3.2.7 Limited disease awareness

**Figure 8:**
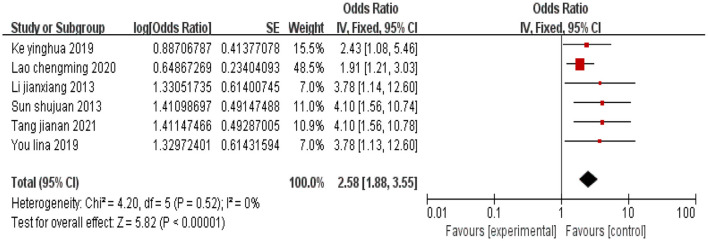
Forest plot of the impact of limited disease awareness on depression in cervical cancer patients.

A total of six studies were included, and there was no heterogeneity among the studies (*I*^2^ = 0%). Using a fixed-effects model, the combined effect size indicated that limited awareness of the disease in cervical cancer patients is associated with depression (*P* < 0.05), as shown in Figure 8.

#### 3.2.8 Hysterectomy

**Figure 9:**
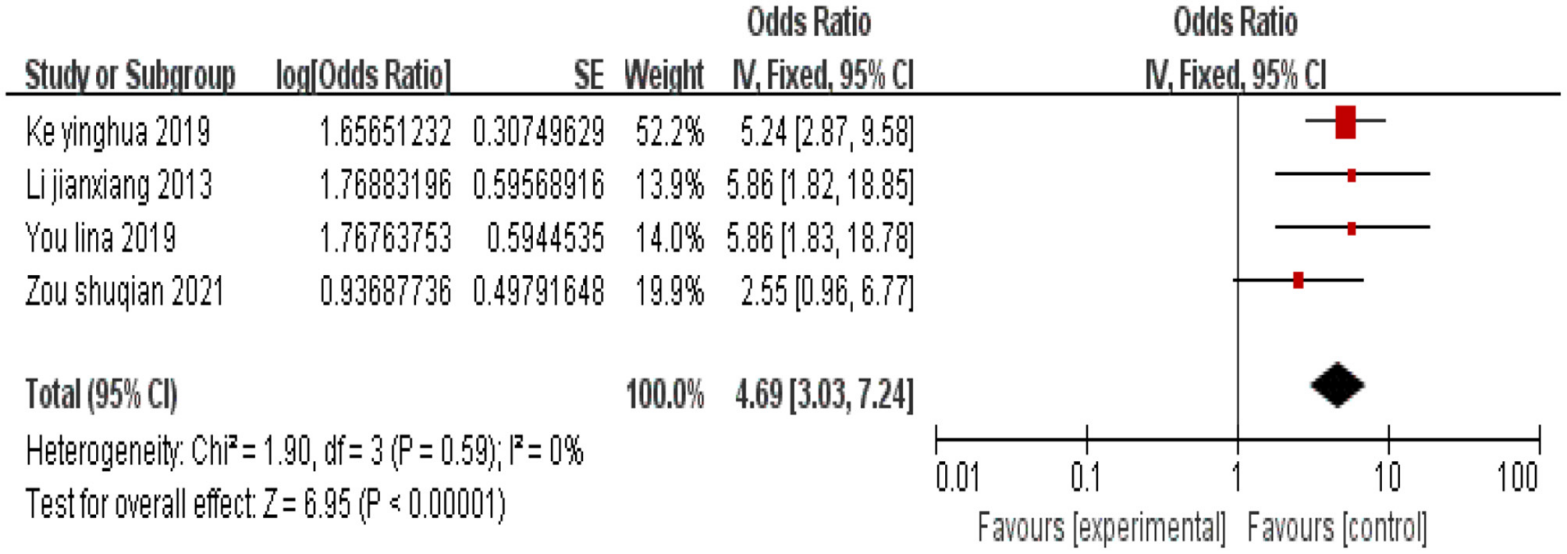
Forest plot of the impact of hysterectomy on depression in cervical cancer patients.

A total of four studies were included, and there was no heterogeneity among the studies (*I*^2^ = 0%). Using a fixed-effects model, the combined effect size indicated that undergoing hysterectomy is associated with depression in cervical cancer patients (*P* < 0.05), as shown in Figure 9.

### 3.3 Subgroup analysis

Due to the significant heterogeneity observed in the analysis of certain influencing factors, subgroup analysis is warranted to further explore the sources of this heterogeneity. Subgroup analyses stratified by different depression assessment scales revealed that for the factor “Inter-household Monthly Income Disparity” subgroup analysis significantly reduced heterogeneity. However, for the factor “Tumor Stage (Advanced Stage)” while heterogeneity decreased, it remained substantial, suggesting that the type of depression assessment scale may contribute to heterogeneity for both these factors. For the factor “Low Educational Attainment” heterogeneity was not reduced; therefore, differences in the depression scales are unlikely to be the source of its heterogeneity. Regarding the factor “age ≥45 years” subgroup analysis could not be performed due to an insufficient number of included studies. The heterogeneity results for each subgroup are presented in Table 4, and the effect size estimates for each subgroup are shown in Supplementary File 1.

**Table 4:** Subgroup analysis.

**Subgroup analysis based on the different etiologies of infertility**	**Number of articles**	***I* ^2^**
**Inter-household monthly income disparity**		
SDS	7	2%
HADS	2	0%
BDI	1	/
HAMD	2	0%
**Tumor stage (advanced stage)**		
SDS	2	78%
HADS	1	/
BDI	2	74%
HAMD	1	/
**Low educational attainment**		
SDS	6	71%
HADS	1	/
BDI	1	/

### 3.4 Publication bias analysis

A bias test was conducted on the influencing factors with a literature count of ≥10 in this study. The influencing factor identified with a literature count of ≥10 was the disparity in monthly family income; hence, a bias test was performed on this factor. Begg's test was conducted on the 12 included studies, yielding a *P*-value of 0.732 >0.05, indicating that there is no publication bias associated with the disparity in monthly family income.

### 3.5 Sensitivity analysis

The reliability of the results was assessed by observing the magnitude of differences between the fixed-effect model and the random-effect model data. The analysis revealed no essential differences between the two models for each influencing factor, suggesting that the results are stable and reliable, as shown in Table 5.

**Table 5:** Sensitivity analysis.

**Risk factors**	**Fixed effect model**	***P-*value**	**Random effect model**	***P-*value**
Low education attainment	3.15 [2.40,4.13]	< 0.01	3.25 [2.02,5.22]	< 0.01
Age ≥45 years	1.39 [1.22,1.59]	< 0.01	1.67 [1.09, 2.55]	0.02
Inter-household monthly income disparity	2.15 [1.80,2.57]	< 0.01	3.06 [1.87, 5.00]	< 0.01
Tumor stage (advanced stage)	1.57 [1.28,1.92]	< 0.01	1.99 [1.28, 3.11]	< 0.01
Low social support	2.48 [1.95,3.16]	< 0.01	2.48 [1.94, 3.17]	< 0.01
Moderate to severe pain	2.86 [1.76,4.65]	< 0.01	2.86 [1.76, 4.65]	< 0.01
Limited disease awareness	2.58 [1.88,3.55]	< 0.01	2.58 [1.88, 3.55]	< 0.01
Hysterectomy	4.69 [3.03,7.24]	< 0.01	4.69 [3.03, 7.24]	< 0.01

## 4 Discussion

This study include a total of 15 high-quality articles. The results of the meta-analysis indicated that cervical cancer patients with low educational attainment (OR = 3.25, 95% CI: 2.02–5.22), age ≥45 years (OR = 1.67, 95% CI: 1.09–2.55), inter-household monthly income disparity (OR = 3.06, 95% CI: 1.87–5.00), advanced tumor stage (OR = 1.99, 95% CI: 1.28–3.11), low social support (OR = 2.48, 95% CI: 1.95–3.16), moderate to severe pain (OR = 2.86, 95% CI: 1.76–4.65), limited disease awareness (OR = 2.58, 95% CI: 1.88–3.55), and undergone hysterectomy (OR = 4.69, 95% CI: 3.03–7.24) are more likely to experience depression.

Subgroup analyses stratified by different depression assessment scales were performed on factors exhibiting significant heterogeneity. The results revealed that heterogeneity was reduced for the factors “inter-household monthly income disparity” and “tumor stage (advanced stage)” suggesting that the variation in depression assessment scales may be a source of heterogeneity for these two factors. Conversely, for the factor “low educational attainment” heterogeneity was not reduced. Therefore, differences in the depression scales are unlikely to be the source of its heterogeneity, and the source remains unidentified based on the current evidence.

### 4.1 Low educational attainment and limited disease awareness

The results of this meta-analysis indicate that low educational attainment and insufficient disease awareness are risk factors for depression in cervical cancer patients. Educational attainment serves as an objective indicator of a patient's knowledge base; those with lower educational attainment have limited understanding of disease-related knowledge and weaker self-learning abilities. This not only restricts their comprehension and adherence to treatment plans but may also exacerbate their physical and mental burden due to cognitive biases (29). Scholar Song (10) pointed out that having an education attainment below junior high school is a relevant factor for post-operative depressive symptoms in cervical cancer patients. A meta-analysis based on 40 studies in China revealed that participants with the lowest educational attainment or those from rural areas had a significantly higher prevalence of moderate depression (30). Therefore, it is crucial to timely disseminate disease knowledge to patients to effectively improve their adverse emotional states.

### 4.2 Age ≥45 years

An age of 45 years or older is a risk factor for depression in cervical cancer patients, consistent with the findings of Lao and Wu (22). Although some studies (31) investigated psychological distress among terminal cancer patients across different age groups, and they indicate that younger patients bear heavier psychological burdens due to multiple social responsibilities (such as care giving, child-rearing, and occupational stress), older patients typically exhibit more pronounced depressive states due to physiological decline and concerns about prognosis. As age increases, patients may develop multiple chronic diseases, experience a decline in physical function, and exhibit reduced treatment tolerance, thereby increasing the risk of depressive symptoms (32, 33). Therefore, clinical interventions should be stratified by age to provide targeted psychological support, enhance treatment adherence, alleviate mental stress, and ultimately improve the quality of life for patients.

### 4.3 Inter-household monthly income disparity and low social support

Inter-household monthly income disparity is a risk factor for depression in cervical cancer patients. Zhou et al. (34) found that patients with low annual family income have a higher risk of depression, with a per capita monthly income of less than 1,000 yuan being significantly associated with post-operative depression in thyroid cancer patients (*P* < 0.05) (35). Economically disadvantaged patients may experience psychological fears, financial burdens, and uncertainties about future planning during diagnosis and treatment, making them more susceptible to depression (36–38). Therefore, it is recommended to alleviate patients' financial burdens through educating patients and their families about medical insurance policies, considering adjustments to medication brands (e.g., using more affordable alternatives), assisting with applications for medical financial aid. At the same time, excessive or unnecessary medical interventions should be avoided.

Insufficient social support also poses a risk for depression. A lack of social support can undermine patients' psychological resilience, leading to a lack of confidence in treatment outcomes (39). Adequate social support can enhance patients' psychological resilience, strengthen life beliefs, and divert attention from distress. Receiving care and support from others during difficult times can effectively reduce negative emotions such as anxiety and depression (40). Hence, medical staff should encourage patients to maintain good communication and connection with family, friends, or healthcare professionals, ensuring they can receive adequate support and assistance during treatment and recovery.

### 4.4 Advanced tumor stage and moderate to severe pain

An advanced tumor stage significantly increases the risk of depression. Tosic et al. (41) study found that higher tumor stages correlate with more intense psychological stress responses in patients. Patients with advanced cervical cancer are prone to a decline in immune function and the exacerbation of negative emotions due to high treatment intensity, uncertainty regarding prognosis, and impaired physical function (42, 43). It is advisable to implement multidisciplinary collaborative care for late-stage patients, focusing on individualized intervention plans for adverse reactions such as cancer pain and bone marrow suppression.

Moderate to severe pain is an important trigger for depression. You and You (19) found that moderate to severe pain significantly contributes to depression in cervical cancer patients. Cancer-related pain, whether from the disease or treatment, often persists throughout therapy, causing substantial psychological stress that impairs recovery and quality of life (44). A patient-centered comprehensive pain management strategy (such as medication optimization combined with psychological desensitization) is recommended to alleviate the emotional chain reactions triggered by pain.

### 4.5 Hysterectomy

Hysterectomy is an independent risk factor for post-operative depression in cervical cancer patients (24). The uterus, as a core structure of the female reproductive system, may lead to identity crises regarding gender and mourning the loss of reproductive function following its removal (45). Furthermore, surgery-induced ovarian dysfunction or hormonal fluctuations may trigger emotional instability. It is suggested that pre-operative psychological assessments and educational interventions be conducted, followed by cognitive-behavioral interventions post-operatively to assist patients in rebuilding psychological adaptation, enhancing treatment tolerance, and improving quality of life.

## 5 Conclusion

Psychological interventions have been confirmed by several studies to effectively alleviate depressive symptoms in cancer patients. For example, cognitive behavioral therapy has been shown to reduce psychological distress by helping patients change negative thinking patterns and develop positive coping strategies (46). Supportive psychotherapy enhances patients' psychological resilience by providing emotional support and problem-solving skills (47). Additionally, psychoeducational interventions have been validated in cancer patients, showing significant improvements in both depressive symptoms and fatigue (48). Targeted interventions are necessary for addressing the risk factors mentioned in the text (e.g., low income). For patients with low income and insufficient social support, relevant administrative legislation can be promoted to establish financial aid programs, while strengthening psychological support for patients and their families to alleviate economic and emotional pressures. For patients with low education, disease education programs should be implemented to enhance their understanding of the condition and help them acquire knowledge on treatment and self-management, thereby reducing the psychological burden caused by cognitive limitations. These interventions not only effectively alleviate depressive symptoms in patients but also contribute to improving treatment adherence and quality of life, while reducing the negative impact of the disease on mental health.

The formulation and implementation of medical policies, although not easily noticeable, have a significant psychological impact on cancer patients during their treatment process. The existence of such policies effectively ensures that cancer patients can access comprehensive treatment in a more convenient and cost-effective manner. Research indicates that, despite the high demand for psychological treatment among cancer survivors, many are unable to access mental health services due to financial constraints (49). This situation may exacerbate the dual burden of cancer and psychological disorders. As a result, a series of corresponding policies have been introduced, such as the Mental Health Parity and Addiction Equity Act (MHPAEA), which aims to reduce economic barriers to mental health services. The American Cancer Society also recommends that if patients cannot afford psychological therapy, they should actively seek free or low-cost psychosocial support services provided by hospitals or clinics. These policies are particularly important for low-income patients and those with significant psychological distress. During clinical treatment, healthcare providers should closely monitor patients' emotional changes, offer timely psychological guidance, and provide relevant disease knowledge. In case of severe adverse reactions, targeted intervention measures should be developed in advance. Furthermore, healthcare providers should collaborate with family members to offer comprehensive care and emotional support to patients, helping them build confidence in their recovery, thereby improving treatment adherence and outcomes, and ultimately enhancing the patients' quality of life and prolonging survival.

While the results of this study are reliable, potential confounders were adjusted for and controlled for in the included studies, certain limitations exist. First, this study is retrospective, with data sourced from medical records and questionnaires, which may introduce bias. Second, the number of studies included for some influencing factors is relatively small. Therefore, future multicenter, large-sample prospective studies are needed for further validation. Thirdly, there is evidence of considerable heterogeneity in certain findings of this study, which may arise from factors including the geographical distribution of study populations, variations in research design, and characteristics of samples.

In summary, low educational attainment, limited disease awareness, age ≥45 years, inter-household monthly income disparity, low social support, advanced tumor stage, moderate to severe pain, and undergoing hysterectomy are risk factors for depression in cervical cancer patients. Therefore, it is essential to address the influencing factors of depression in cervical cancer patients through clinical and familial approaches, implementing individualized interventions to improve patients' negative psychological states, alleviate depressive emotions, and ultimately enhance their quality of life and survival duration.

## Data Availability

The original contributions presented in the study are included in the article/Supplementary material, further inquiries can be directed to the corresponding author.

## References

[1] China Anti-Cancer Association Gynecologic Oncology Committee. Guidelines for diagnosis and treatment of cervical cancer (2021 Edition). Chin Oncol J. (2021) 31:474–89. 10.19401/j.cnki.1007-3639.2021.06.06

[2] Chinese Society for Colposcopy and Cervical Pathology of China Healthy Birth Science Association; Chinese Society of Gynecological Oncology, Chinese Medical Association . Guidelines for cervical cancer screening in China II. Prog Obstet Gynecol. (2025) 34:1–9. 10.13283/j.cnki.xdfckjz.2025.01.001

[3] Bray F Laversanne M Sung H Ferlay J Siegel RL Soerjomataram I . Global cancer statistics 2022: GLOBOCAN estimates of incidence and mortality worldwide for 36 cancers in 185 countries. CA Cancer J Clin. (2024) 74:229–63. 10.3322/caac.21834 38572751

[4] Yao Y Sun K Zheng R. Interpretation and analysis of the Global Cancer Statistics Report 2022:a comparison between China and the world. Chin J Bases Clin Gen Surg. (2024) 31:769–80. 10.7507/1007-9424.202406046

[5] Zhang L. Relationship between Symptom Clusters and Quality Of Life in Patients With Cervical Cancer Undergoing Concurrent Chemoradiotherapy: The Mediation Effect Of Illness Perception. Jinan: Shandong University (2021). 10.3389/fpsyt.2021.807974

[6] Li J Pan Q Ban T. Risk factors analysis on depression psychological of patients with cervical cancer. Hebei Med. (2013) 19:1619–21. 10.3969/j.issn.1006-6233.2013.11.007

[7] Yu X Fu B Dong L Li X Cao L Fu Z. Investigation on coenitive emotion regulation and anxiety as well as depression in patients with cervical cancer accepting postoperative chemotherapy and their correlation analysis. Chin J Woman Child Health Res. (2018) 29:302–5. 10.3969/j.issn.1673-5293.2018.03.014

[8] Jiang X Tang H Chen T. Epidemiology of gynecologic cancers in China. J Gynecol Oncol. (2018) 29:e7. 10.3802/jgo.2018.29.e7 29185265 PMC5709533

[9] Xiaoping D. Research on the Predictive Model and Cognitive Behavior Intervention Programme for Depression among Cervical Cancer Patients. Huazhong University of Science and Technology (2023).

[10] Satin JR Linden W Phillips MJ. Depression as a predictor of disease progression and mortality in cancer patients: a meta-analysis. Cancer. (2009) 115:5349–61. 10.1002/cncr.24561 19753617

[11] Kiecolt-Glaser JK Glaser R. Depression and immune function: central pathways to morbidity and mortality. J Psychosom Res. (2002) 53:873–6. 10.1016/S0022-3999(02)00309-4 12377296

[12] Song H. Related influencing factors and nursing countermeasures for depressive symptoms in postoperative cervical cancer patients. Modern Nurse. (2021) 28:22–4. 10.19791/j.cnki.1006-6411.2021.07.009

[13] Zhu Y Zhu Q Qin J. Investigation of the depression status of patients with cervical cancer during the radiotherapy and analysis of its influence factors. Chin J Fam Plann. (2024) 32:1247–51.

[14] Wang Y Dong X Huang D Deng K Hu B. Depression and its influencing factors in patients with cervical cancer undergoing concurrent radio-therapy and chemotherapy. J Int Psychiatry. (2020) 47:599–601, 605. 10.13479/j.cnki.jip.2020.03.056

[15] Yang Y. Depression and Quality of Life and Correlation Analysis Inpatients with Cervical Cancer during Concurrent Chemoradiotherapy. Anhui: Anhui Medical University (2018).

[16] He L. Investigation on depression and anxiety status and influencing factors in cervical cancer patients undergoing postoperative chemotherapy. Mater Child Health Care Chin. (2022) 37:3427–30. 10.19829/j.zgfybj.issn.1001-4411.2022.18.036

[17] Tang J Luo W. Factors influencing anxiety and depression in young and middle-aged cervical cancer patients after surgery and corresponding nursing strategies. Int J Nurs. (2021) 40:2148–52. 10.3760/cma.j.cn221370-20200420-00617

[18] Sun S. Study on Prevalence and Influencing Factors of Depressive and Anxiety Symptoms in Postoperative Patients with Cervical Cancer. Hunan: Central South University (CSU) (2013).

[19] You L You J. Negative emotion survey and risk factor analysis in patients with cervical cancer. Doctor. (2019) 4:24–6. 10.3760/cma.j.issn.1673-4351.2020.02.010

[20] Ke Y Yu M Mai W Liu C. Analysis of risk factors of depression in patients with cervical cancer and its correlation with TCM constitution. Shandong J Tradit Chin Med. (2019) 38:766–9. 10.16295/j.cnki.0257-358x.2019.08.013

[21] Wang X Wang S-J. Analysis of depression and anxiety status and related factors in patients receiving postoperative chemotherapy for cervical cancer. Chin J Pract Gynecol Obstet. (2019) 35:1035–8. 10.19538/j.fk2019090120

[22] Lao C Wu H. Investigation of psychological status and related factors in cervical cancer patients undergoing postoperative chemotherapy. Zhejiang J Int Tradit Chin West Med. (2020) 30:771–4. 10.3969/j.issn.1005-4561.2020.09.024

[23] Zhu J Ma Y Sun X. The risk factors of depression in patients undergoing cervical cancer radical surgery. J Int Psychiatry. (2024) 51:1579–82. 10.13479/j.cnki.jip.2024.05.024

[24] Zou S Zhan L Li K Zou SQ Zhan LJ Li KJ . Establishment of the nomogram model for predicting the risk factors of depression after cervical cancer surgery. J Mod Med Health. (2021) 37:3607–10, 3615. 10.3969/j.issn.1009-5519.2021.21.004

[25] Kim SH Kang S Kim YM Kim BG Seong SJ Cha SD . Prevalence and predictors of anxiety and depression among cervical cancer survivors in Korea. Int J Gynecol Cancer. (2010) 20:1017–24. 10.1111/IGC.0b013e3181e4a704 20683411

[26] Stang A. Critical evaluation of the Newcastle-Ottawa scale for the assessment of the quality of nonrandomized studies in meta-analyses. Eur J Epidemiol. (2010) 25:603–5. 10.1007/s10654-010-9491-z 20652370

[27] Zeng X Liu H Chen X Zhang Y Wang Y Zhang C . Meta-analysis series part IV: quality assessment tools for observational studies. Chin J Evid Based Cardiovasc Med. (2012) 4:297–9. 10.3969/j.1674-4055.2012.04.004

[28] Zhou Y Gu Y Hu Y Xing W. The Joanna Briggs institute critical appraisal tools for use in systematic review. Prevalence study and analytical cross sectional study. J Nurs Train. (2018) 33:219–22. 10.16821/j.cnki.hsjx.2018.05.005

[29] Hong L. Analysis of suicide risk in patients with depression and its related influencing factors. Heilongjiang Med J. (2024) 48:2194–6. 10.3969/j.issn.1004-5775.2024.18.005

[30] Zhao YJ Jin Y Rao WW Zhang Q-E Zhang L Jackson T . Prevalence of major depressive disorder among adults in china: a systematic review and meta-analysis. Front Psychiatry. (2021) 12:659470. 10.3389/fpsyt.2021.659470 34168579 PMC8219051

[31] Zhang H Zheng S. Comparative analysis of psychological distress in advanced cancer patients across different age groups. Modern Nurse. (2022) 29:41–3. 10.19793/j.cnki.1006-6411.2022.18.012

[32] Wei Q Bi Q Hu C Li B Wang Z Chen X Qin Y. Relationship between hope and depression among patients undergoing chemotherapy after radical resection of rectal neoplasms. Anhui Med Pharm J. (2019) 23:1975–9.

[33] Chen X Zheng Z Du C. Effects of psychological intervention on depression and quality of life in breast cancer patients undergoing adjuvant chemotherapy. J Chin Oncol. (2018) 24:1201–5. 10.11735/j.issn.1671-170X.2018.12.B012

[34] Zhou X Wei S Lu M. Analysis of depression influencing factors in cancer patients based on lasso-logistic regression and random forest models. Anhui Med J. (2024) 45:1177–82. 10.3969/j.issn.1000-0399.2024.09.021

[35] Fang L Yang Y Feng X. Wang J. Influencing factors and intervention strategies of postoperative anxiety, depression and psychosocial adaptation status in patients with thyroid cancer. J Clin Nurs Pract. (2025) 11:129–32. 10.11997/nitcwm.202501033

[36] Riedl D Schüßler G. Factors associated with and risk factors for depression in cancer patients - A systematic literature review. Transl Oncol. (2022) 16:101328. 10.1016/j.tranon.2021.101328 34990907 PMC8741617

[37] Pang Y He Y Wang Y Lu Y Jiang Y Song L . Insomnia in the elderly adults with advanced cancer: a national multi-center survey. Chin J Multi Organ Dis Elder. (2022) 21:826–30. 10.11915/j.issn.1671-5403.2022.11.177

[38] Wang X Wang J Li J Liu H. Wang J. Investigation of influencing factors for depressive mood in patients with advanced gastric cancer and its relationship with chemotherapy tolerance. Pract Prevent Med. (2022) 29:1364–8. 10.3969/j.issn.1006-3110.2022.11.020

[39] Liu N Yang Y Wang W Chu H Yang X Zhou J . Relationship between perceived social support and depression in cervical cancer patients during chemotherapy: the intermediary mechanism of rumination. J Harbin Med Univ. (2021) 55:557–60. 10.3969/j.issn.1000-1905.2021.05.024

[40] Li H Lyu M Wang A Yin Y Zhang J Li P. Social support and life satisfaction in women with cervical cancer: a serial multiple mediation model. Cancer Nurs. (2024) 47:64–71. 10.1097/NCC.0000000000001146 36322694

[41] Tosic Golubovic S Binic I Krtinic D Djordjevic V Conic I Gugleta U . Risk factors and predictive value of depression and anxiety in cervical cancer patients. Medicina. (2022) 58:507. 10.3390/medicina58040507 35454346 PMC9027265

[42] Chen X He X. Analysis of psychological distress and its influencing factors in postoperative lung cancer patients. Chin J Mod Nurs. (2021) 27:3318–22. 10.3760/cma.j.cn115682-20210226-00881

[43] Wu D Li F Lin L. Analysis of depressive status, cancer-related fatigue, and their influencing factors in breast cancer patients. J Clin Nurs Pract. (2024) 10:129–31. 10.11997/nitcwm.202406039

[44] Swarm RA Paice JA Anghelescu DL Are M Bruce JY Buga S . Adult cancer pain, version 3.2019, NCCN clinical practice guidelines in oncology. J Natl Compr Canc Netw. (2019) 17:977–1007. 10.6004/jnccn.2019.0038 31390582

[45] Lee KS Vaillancourt T. Longitudinal associations among bullying by peers, disordered eating behavior, and symptoms of depression during adolescence. JAMA Psychiatry. (2018) 75:605–12. 10.1001/jamapsychiatry.2018.0284 29641816 PMC6137525

[46] Zhang L Liu X Tong F Zou R Peng W Yang H . Cognitive behavioral therapy for anxiety and depression in cancer survivors: a meta-analysis. Sci Rep. (2022) 12:21466. 10.1038/s41598-022-25068-7 36509786 PMC9744858

[47] Sharpley CF Christie DR Bitsika V Miller BJ. Trajectories of total depression and depressive symptoms in prostate cancer patients receiving six months of hormone therapy. Psychooncology. (2017) 26:60–6. 10.1002/pon.4100 26857160

[48] Wang Y Lin Y Chen J Wang C Hu R Wu Y. Effects of Internet-based psycho-educational interventions on mental health and quality of life among cancer patients: a systematic review and meta-analysis. Support Care Cancer. (2020) 28:2541–52. 10.1007/s00520-020-05383-3 32179998

[49] Arega MA Dee EC Muralidhar V Nguyen PL Franco I Mahal BA . Psychological distress and access to mental health services among cancer survivors: a national health interview survey analysis. J Gen Intern Med. (2021) 36:3243–5. 10.1007/s11606-020-06204-3 32935313 PMC8481450

