# Supplementary figures and images for: Meta-analysis of factors influencing depression in cervical cancer patients
Source: Front Public Health. 2025 Nov 3;13:1657690. doi: 10.3389/fpubh.2025.1657690 (PMC12620220; doi:10.3389/fpubh.2025.1657690)

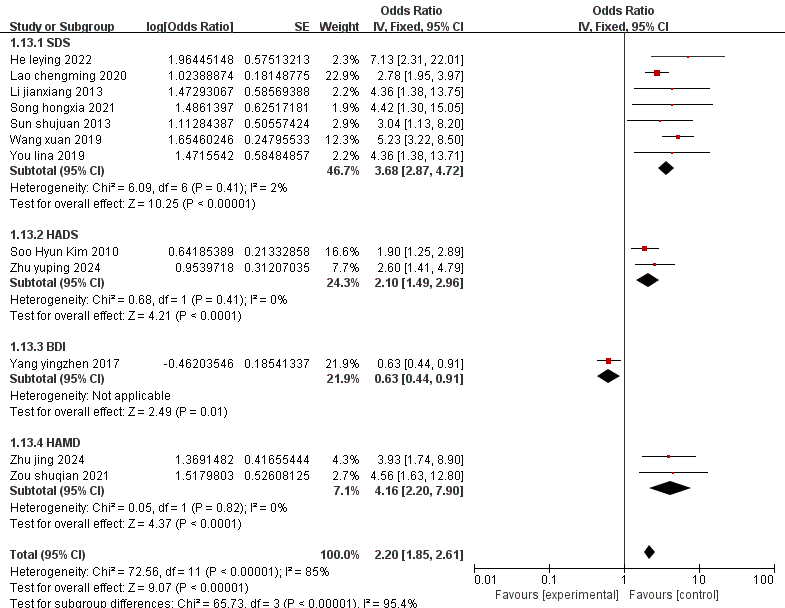

Supplement: Supplementary file 1 [file Supplementary_file_1.zip › Supplementary Figure/Family monthly income.png]

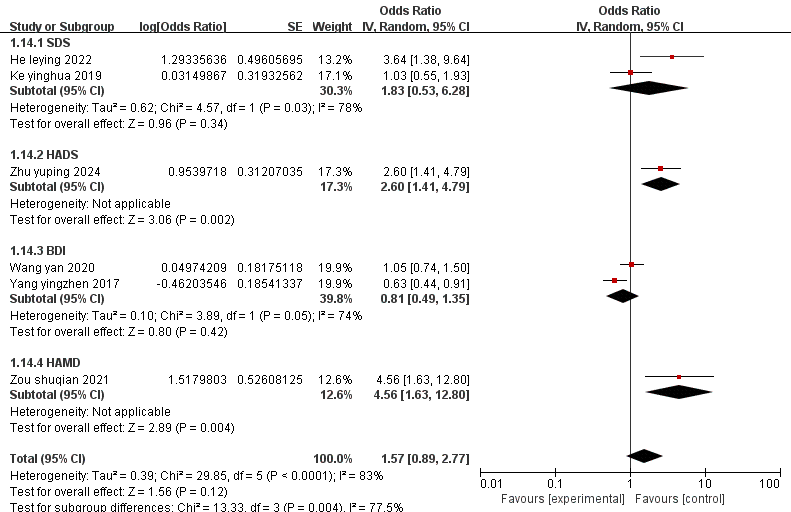

Supplement: Supplementary file 1 [file Supplementary_file_1.zip › Supplementary Figure/neoplasm staging.png]

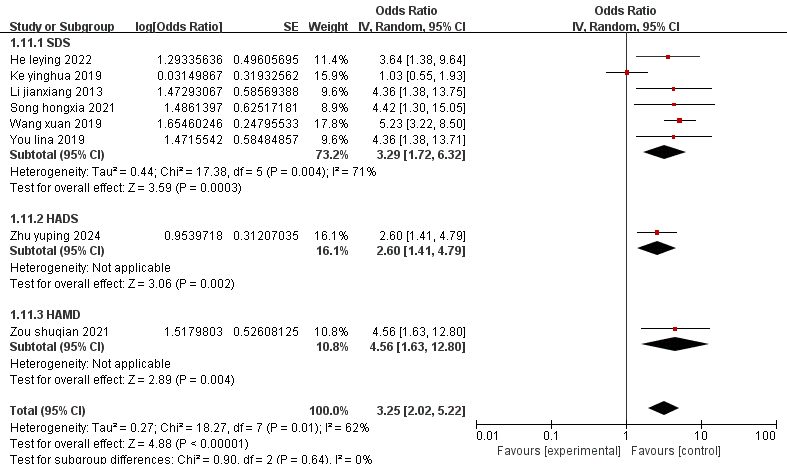

Supplement: Supplementary file 1 [file Supplementary_file_1.zip › Supplementary Figure/standard of culture.png]
